# Variations in the Gut Microbiota in Breast Cancer Occurrence and Bone Metastasis

**DOI:** 10.3389/fmicb.2022.894283

**Published:** 2022-05-26

**Authors:** Yu Wenhui, Xie Zhongyu, Chen Kai, Cai Zhaopeng, Li Jinteng, Ma Mengjun, Su Zepeng, Che Yunshu, Wang Peng, Wu Yanfeng, Shen Huiyong

**Affiliations:** ^1^Department of Orthopedics, The Eighth Affiliated Hospital of Sun Yat-sen University, Shenzhen, China; ^2^Breast Tumor Center, Sun Yat-sen Memorial Hospital, Sun Yat-sen University, Guangzhou, China; ^3^Center for Biotherapy, The Eighth Affiliated Hospital of Sun Yat-sen University, Shenzhen, China

**Keywords:** gut microbiota, bone metastasis (BM), breast cancer neoadjuvant therapy, 16S rRNA sequencing, PICRUSt (functional genes)

## Abstract

Breast cancer is the most common cancer in women and the second most common cancer overall. Although advancements in the early diagnosis and therapy of breast cancer have occurred in recent years, the prognosis of breast cancer bone metastasis remains poor and this type of cancer is rarely cured. The gut microbiota is indispensable for internal homeostasis and regulates various biological processes. Understanding the gut microbiota profiles in normal controls (NCs), breast cancer patients with no metastasis (BNs), and breast cancer patients with bone metastasis (BMs) may shed light on the development of diagnostic and therapeutic targets for breast cancer and bone metastasis. We comprehensively analyzed the gut microbiota from NCs, BNs, and BMs and found that the community diversity decreased in the order of NCs, BNs, and BMs. *Streptococcus*, *Campylobacter* and Moraxellaceae showed higher abundances in BNs and BMs than in NCs. The lack of *Megamonas* and *Akkermansia* in the BM compared with those in the NC and BN groups was considered related to bone metastasis. Additionally, based on the distinct gut microbiota profiles, we predicted that lipid transportation and metabolism, as well as folate biosynthesis, participate in breast cancer occurrence and that steroid hormone biosynthesis influences bone metastasis. Our study demonstrated that variations in gut microbiota are associated with breast cancer occurrence and bone metastasis, providing attractive targets to develop therapeutic and diagnostic methods.

## Introduction

Breast cancer is the most prevalent malignancy among women and the second most common cancer overall. Approximately 1.7 million diagnosed cases and 52 thousand deaths occurred in 2012 worldwide (Ferlay et al., 2015), causing considerable physical and financial burdens. Twenty to thirty percent of breast cancer patients develop metastases, which are responsible for 90% of deaths related to breast cancer (Allemani et al., 2018). In breast cancer patients, metastases most frequently appear in the bone (Brook et al., 2018), and the originally immobilized growth factors, such as transforming growth factor-beta, are released after osteolytic destruction by breast cancer, completing the vicious cycle of bone metastasis (Ma et al., 2020). According to a recent study, the overall survival from bone metastasis diagnosis is 40 months (Kuchuk et al., 2013), emphasizing the need to explore novel prevention and treatment modalities against bone metastases in breast cancer.

The gut microbiota colonizing the human intestine is considered indispensable for the homeostasis of organ development (Mayer et al., 2015), the immune response (D’Amelio and Sassi, 2018), and metabolism (Morrison and Preston, 2016), among other functions. However, the disruption of the gut microbiota contributes to the development of various diseases, such as cardiovascular diseases, autism, type 2 diabetes, inflammatory bowel disease, and malignancy. Growing evidence indicates the relationship between gut microbiota and breast cancer occurrence. A recent study revealed that the distinct gut microbiota in postmenopausal breast cancer patients might be responsible for the occurrence of breast cancer (Zhu et al., 2018). The prognosis and therapeutic effect of breast cancer were also linked to the gut microbiota according to recent reports (McKee et al., 2021; Terrisse et al., 2021). Several studies have demonstrated that the gut microbiota regulates the metastasis of melanoma (Matson et al., 2018) and colorectal cancer (Li et al., 2019), as well as lung metastasis of breast cancer (Quail et al., 2017). Despite the increased attention concerning the role of the gut microbiota in breast cancer and cancer metastasis, whether or which gut microbiota contribute to the bone metastasis of breast cancer remains unknown.

The bone microenvironment contains various cell types, including immune cells, megakaryocytes, and myeloid cells derived from myeloid lineage cells, as well as osteoblasts and osteoclasts. Disruption of bone microenvironment homeostasis results in a distorted immune response and bone remodeling, leading to bone metastasis. The gut microbiota regulates bone homeostasis through nutrient absorption, immune regulation, and direct translocation (Hernandez et al., 2016). Li et al. (2016) demonstrated that sex steroid–depleted mice raised in germ-free (GF) conditions are protected against trabecular bone loss and that microbial recolonization of GF mice restores the capacity of sex steroid deficiency to induce bone loss. Sjogren et al. (2012) revealed that the absence of gut microbiota leads to increased bone mass associated with a reduced number of osteoclasts (OCLs) in trabecular bone and a decreased frequency of CD4 + T cells and OCL precursor cells in bone marrow, indicating the influence of the gut microbiota on the bone microenvironment. Considering the simultaneous effects of the gut microbiota on cancer occurrence, metastasis, and bone microenvironment, we postulated that the gut microbiota plays a crucial role in the bone metastasis of breast cancer.

We conducted a comprehensive analysis of fecal samples from normal controls (NCs), breast cancer patients with no metastasis (BNs), and breast cancer patients with bone metastasis (BMs). As the first study to report the relationship between gut microbiota and breast cancer metastasis, the altered gut microbiota and biological processes involved in breast cancer occurrence and bone metastasis were first identified, which could provide new insight into treating breast cancer bone metastasis by developing new treatment combining gut microbiota interfering, such as antibiotics or probiotics administration, with the regular treatments to increased the chance for curing advanced breast cancer.

## Materials and Methods

### Participant Recruitment and Sample Collection

This study was approved by the ethics committee of Sun Yat-sen Memorial Hospital, Sun Yat-sen University. All fecal samples were collected from August 2019 to February 2020. Subjects with gastrointestinal diseases, chronic diseases that required long-term medication that may alter the gut microbiota, and breast cancer patients who developed metastasis at sites other than bone were excluded. All the patients were free of radiotherapy or chemotherapy for 8 weeks, antibiotic administration for 4 weeks, or surgery for 6 months. The recruited patients were hospitalized for at least two weeks and were provided with the same diet to minimize the influence of diet on the structure of the gut microbiota during fecal sample collection. All the participants who agreed to donate fecal samples provided written informed consent. Breast cancer patients were diagnosed by pathological examination at the Breast Tumor Center of Sun Yat-sen Memorial Hospital, and normal controls were recruited from the medical examination center of Sun Yat-sen Memorial Hospital. We collected 25 NC, 32 BN, and 22 BM fecal samples. The fecal samples were freshly collected and frozen in liquid nitrogen, transferred to the laboratory and stored at –80°C until extraction.

### DNA Extraction and Sequencing

Microbial community DNA was extracted using a MagPure Stool DNA KF kit B (Magen, China) following the manufacturer’s instructions. DNA was quantified using a Qubit Fluorometer and a Qubit dsDNA BR Assay kit (Invitrogen, United States), and the quality was checked by running aliquots on a 1% agarose gel electrophoresis.

The sequencing procedure was performed as previously described with some modifications (Caporaso et al., 2012). Variable region V4 of the bacterial 16S rRNA gene was amplified using degenerate polymerase chain reaction (PCR) primers 515F (5′-GTGCCAGCMGCCGCGGTAA-3′) and 806R (5′-GGACTACHVGGGTWTCTAAT-3′). Both the forward and reverse primers were tagged with Illumina adapter, pad and linker sequences. PCR enrichment was performed in a 50-μL reaction containing 30 ng of the template, the fusion PCR primer, and the PCR master mix. The PCR cycling conditions were as follows: 95°C for 3 min; 30 cycles of 95°C for 45 s, 56°C for 45 s, and 72°C for 45 s; and a final extension for 10 min at 72°C. The PCR products were purified using Agencourt AMPure XP beads and were eluted in the elution buffer. The libraries were qualified using the Agilent Technologies 2100 bioanalyzer. The validated libraries were used for sequencing on the Illumina HiSeq 2500 platform (BGI, Shenzhen, China) following the standard pipelines of Illumina, generating 2 × 250 bp paired-end reads.

### Bioinformatics Analysis

Raw reads were filtered to remove the adapters and low-quality and ambiguous bases, and then paired-end reads were added to the tags using the Fast Length Adjustment of Short reads program (FLASH, v1.2.11) (Magoč and Salzberg, 2011) to obtain the tags. The tags were clustered into operational taxonomic units (OTUs) with a cutoff value of 97% using UPARSE software (v7.0.1090) (Edgar, 2013), and chimera sequences were compared with the Gold database using UCHIME (v4.2.40) (Edgar et al., 2011) for detection. Next, OTU representative sequences were taxonomically classified using Ribosomal Database Project (RDP) Classifier v.2.2 with a minimum confidence threshold of 0.6 and then were trained on the Greengenes database v201305 by QIIME (v1.8.0) (Caporaso et al., 2010). USEARCH_global (Edgar, 2010) was used to compare all the tags back to OTUs to obtain the OTU abundance statistics table of each sample. Alpha and beta diversity indexes were estimated using MOTHUR (v1.31.2) (Schloss et al., 2009) and QIIME (v1.8.0) at the OTU level, and the observed species and Chao and ACE indexes were used to evaluate the alpha diversity. Sample clustering was conducted using QIIME (v1.8.0) based on UPGMA. KEGG and COG functions were predicted using PICRUSt software (Wilkinson et al., 2018). Bar plots and heatmaps of different classification levels were plotted using the R package gplots (v3.4.1). PLS-DA was performed using the R package mixOmics. A GraPhlAn map of species composition was created using GraPhlAn. Significant species or functions were determined by R (v3.4.1) based on the Wilcoxon or Kruskal–Wallis test.

## Results

### Clinical Characteristics of the Included Subjects

To investigate the role of the gut microbiota in the occurrence and metastasis of breast cancer, we collected fecal samples from 25 NCs, 32 BNs, and 22 BMs, and the flat OTU cumulative curve indicated a sufficient sample size to identify the complete microbiome in the fecal samples (Supplementary Figure 1). The average ages of the NC, BN, and BM groups were 54.08 ± 11.17, 52.06 ± 10.95, and 50.77 ± 10.72 years, respectively, without a significant difference (*P* = 0.551). Because some of the included subjects had a history of antibiotic use, we collected the fecal samples 4 weeks after the last antibiotic treatment to exclude the effect of antibiotics on gut microbiota homeostasis, and the proportions of antibiotic use history were not significantly different among the NC, BN and BM groups (*P* = 0.941). No significant differences were found in the proportion of surgery (*P* = 0.593), radiotherapy (*P* = 0.309) and chemotherapy (*P* = 0.122) treatments. Regarding the pathological characteristics, the positive percentages of estrogen receptor (ER), progesterone receptor (PR), and human epidermal growth factor receptor 2 (HER2) also showed no significant difference (*P* = 0.945, 0.087, and 0.522, respectively) between the BN and BM groups. All the patients were free of radiotherapy or chemotherapy for 8 weeks or surgery for 6 months. The recruited patients were hospitalized for at least two weeks and provided with the same diet to minimize the influence of diet on the structure of the gut microbiota during fecal sample collection. The detailed characteristics of the included subjects are shown in Table 1 and Supplementary Table 1, and the detailed statistics for the quality control of 16S rRNA sequencing are shown in Supplementary Table 2.

**TABLE 1:** Clinical features of normal control and breast cancer patients with or without bone metastasis.

Clinical feature	Group			*P* value
	NC	BN	BM	
All patients (n,%)	25 (100)	32 (100)	22 (100)	
Age (mean ± sd)	54.08 ± 11.17	52.06 ± 10.95	50.77 ± 10.72	0.551
**Antibiotics (n,%)**				
Yes	7 (28)	10 (31.3)	6 (27.3)	0.941
No	18 (72)	22 (68.8)	16 (72.7)	
**Surgery (n,%)**				
Yes	–	24 (8)	15 (68.2)	0.583
No	–	8 (12.5)	7 (31.8)	
**Radiotherapy (n,%)**				
Yes	–	4 (12.5)	6 (27.3)	0.309
No	–	28 (87.5)	16 (72.7)	
**Chemotherapy (n,%)**				
Yes	–	15 (46.9)	15 (68.2)	0.122
No	–	17 (53.1)	7 (31.8)	
**ER (n,%)**				
–	–	9 (28.1)	6 (27.3)	0.945
+ / + + / + + +	–	23 (71.9)	16 (72.7)	
**PR (n,%)**				
–	–	22 (68.8)	10 (45.5)	0.087
+ / + + / + + +	–	10 (31.3)	12 (54.5)	
**HER2 (n,%)**				
–	–	9 (28.1)	8 (36.4)	0.522
+ / + + / + + +	–	23 (71.9)	14 (63.6)	

### Microbial Diversity Decreases in the Order of Normal Controls, Breast Cancer Patients With No Metastasis, and Breast Cancer Patients With Bone Metastasis

Alpha diversity represents the microbiome richness and community diversity of the intestine, and disturbed homeostasis of the gut microbiota leads to altered alpha diversity and dysfunction of the gut microbiota, which subsequently contributes to the occurrence and exacerbation of diseases. We first analyzed the alpha diversity of the NC, BN, and BM groups to characterize the general features of the gut microbiota, and the observed species, Chao, and ACE indexes were chosen to evaluate bacterial richness and community diversity.

**FIGURE 1:**
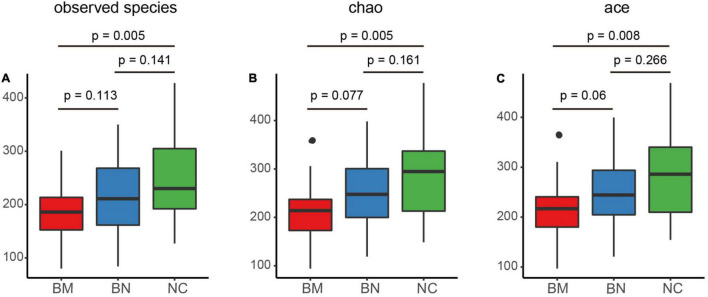
Comparison of the gut microbiota alpha and beta diversity indexes of NCs, BNs, and BMs. **(A–C)** Box plots comparing the alpha diversity of NCs, BNs, and BMs using observed species **(A)**, Chao **(B)** and ACE **(C)** indexes. NCs, normal controls; BNs, breast cancer patients with no metastasis; BMs, breast cancer patients with bone metastasis. P values were calculated using the Kruskal–Wallis test.

Comparing the alpha diversity between the BN and NC groups, decreases were found in the observed species (*P* = 0.141), Chao (*P* = 0.161), and ACE (*P* = 0.266) indexes, but the differences did not reach significance (Figures 1A–C). Additionally, the BM group had decreased values of the observed species (*P* = 0.113), Chao (*P* = 0.077) and ACE (*P* = 0.06) indexes compared with those of the BN group (Figures 1A–C). Although the BN versus NC group and BM versus BN group were not significantly different, the BM group showed significantly decreased observed species compared to the NC group (*P* = 0.005), Chao (*P* = 0.005), and ACE (*P* = 0.008) indexes compared with the NC group (Figures 1A–C). Next, we assessed the difference in alpha diversity among the three groups. Although no significant differences were found between the BN and NC groups and between the BM and BN groups, the Kruskal–Wallis test showed significantly decreased observed species (*P* = 0.018), Chao (*P* = 0.016), and ACE (*P* = 0.024) indexes (Figures 1A–C), revealing the reduced bacterial richness and community diversity in the order of NCs, BNs, and BMs.

### Distinct Profiles in the Gut Microbiota Among the Normal Controls, Breast Cancer Patients With No Metastasis, and Breast Cancer Patients With Bone Metastasis

**FIGURE 2:**
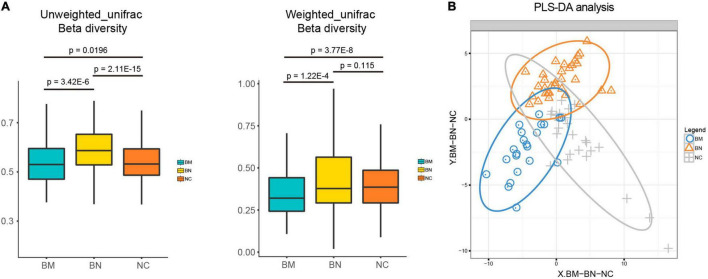
Analyses of the gut microbiota community profiles. **(A)** Box plots comparing the beta diversity of NCs, BNs, and BMs. **(B)** PLS-DA of the distinct gut microbiota community profiles of NCs, BNs, and BMs.

No significant cluster was found among the group in unweighted or weighted UniFrac beta diversity (Supplementary Figure 2), but the box plots showed that beta diversity among the 3 groups was significantly different, except for the comparison between the BN and NC groups in weighted UniFrac beta diversity, indicating the diverse microbial structure among the NC, BN, and BM groups (Figure 2A). Although we revealed decreased bacterial richness and community diversity in the order of NCs, BNs, and BMs, whether the compositional profiles of the three groups differ from each other must be clarified. Partial least squares discriminant analysis (PLS-DA) was performed to confirm the distinct compositions of the gut microbiota among the NC, BN, and BM groups. Although BNs versus NCs and BMs versus BNs showed no significant difference in alpha diversity, they showed distinct compositional profiles according to PLS-DA (Figure 2B). As expected, BMs and NCs also showed a discrepancy in the gut microbiota composition (Figure 2B) because the alpha diversity was significantly different.

### Gut Microbial Composition Analysis of the Fecal Samples

**FIGURE 3:**
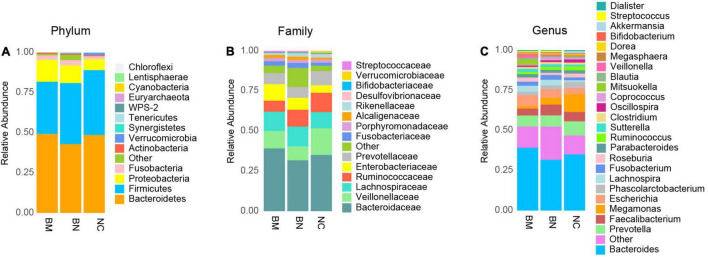
Compositional analysis of the gut microbiota community. **(A)** Compositional analysis of the gut microbiota community in NCs, BNs and BMs at the phylum level. **(B)** Compositional analysis of the gut microbiota community in NCs, BNs, and BMs at the family level. **(C)** Compositional analysis of the gut microbiota community in NCs, BNs, and BMs at the genus level.

To further clarify the composition of the gut microbiota, we analyzed the taxon profiles of the NC, BN and BM groups at the phylum, family and genus levels (Supplementary Table 3). At the phylum level, the top 5 were Bacteroidetes (48.60%, 42.92% and 49.3%, in the order of NCs, BNs, and BMs, respectively), Firmicutes (40.50%, 38.09% and 32.51%) Proteobacteria (6.81%, 11.04% and 13.63%), Fusobacteria (1.85%, 3.19% and 2.74%) and Actinobacteria (0.68%, 0.70% and 0.71%) (Figure 3A). The 5 most abundant families were Bacteroidaceae (35.10%, 31.76% and 39.20%), Veillonellaceae (16.60%, 8.66% and 10.87%), Lachnospiraceae (10.13%, 12.41% and 11.99%), Ruminococcaceae (12.03%, 10.46% and 6.88%), and Enterobacteriaceae (4.72%, 7.46% and 10.32%) (Figure 3B). Among the listed most abundant genera, the 5 most abundant were *Bacteroides* (35.06%, 31.72% and 39.18%), *Prevotella* (8.74%, 6.99% and 6.85%), *Faecalibacterium* (5.87%, 6.71% and 4.30%), *Megamonas* (11.16%, 4.35% and 1.69%) and *Escherichia* (3.73%, 5.25% and 6.61%) (Figure 3C).

### Identification of the Signature Gut Microbiota in Breast Cancer Occurrence and Bone Metastasis

**FIGURE 4:**
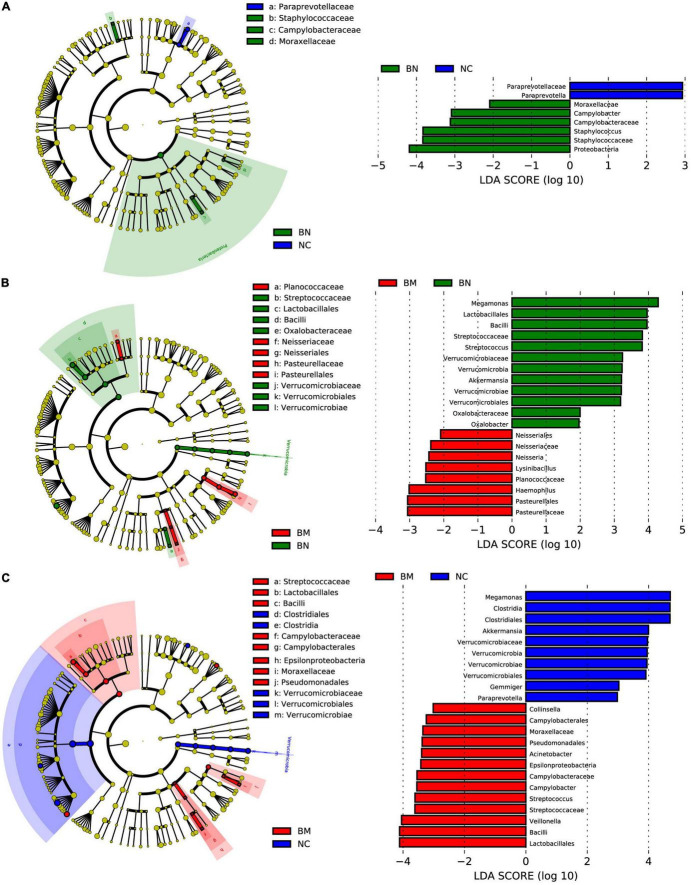
LEfSe analysis identifies signature microbiota constituents that regulate breast cancer occurrence and bone metastasis. **(A)** Cladogram constructed with LEfSe indicating the significantly different taxa between NCs and BNs, and the bar plot shows the LDA score of the different taxa. **(B)** Cladogram constructed with LEfSe indicating the significantly different taxa between BNs and BMs, and the bar plot shows the LDA score of the different taxa. **(C)** Cladogram constructed with LEfSe indicating the significantly different taxa between NCs and BMs, and the bar plot shows the LDA score of the different taxa. LDA scores (log10) > 2 and *P* < 0.05 were considered significantly different.

To identify the significantly different and signature taxa in NCs, BNs, and BMs, linear discriminant effect size (LEfSe) analysis and cladograms were used to elucidate the microbial structure (Supplementary Table 4), and linear discriminant analysis (LDA) was used to estimate the difference in the effect size of each taxon among the NC, BN, and BM groups. Compared with the NC group, the abundances of Proteobacteria, *Staphylococcus*, *Campylobacter*, and Moraxellaceae were significantly higher and those of *Paraprevotella* were lower in the BN group (Figure 4A). Comparing the BM and BN groups, Pasteurellaceae, *Haemophilus*, Planococcaceae, Lysinibacillus, and Neisseria in the BM group and Megamonas, Lactobacillales, Bacilli, *Streptococcus*, *Akkermansia*, and *Oxalobacter* in the BN group were identified as dominant taxa (Figure 4B). As shown by the cladogram and bar plots, compared with the NC group, the gut microbiota of the BM group demonstrated significantly higher levels of Lactobacillales, Bacilli, Veillonella, *Streptococcus*, *Campylobacter*, Epsilonproteobacteria, Acinetobacter, Pseudomonadales, Moraxellaceae, and Collinsella and lower levels of Megamonas, *Clostridia*, *Akkermansia*, Gemmiger, and Paraprevotella (Figure 4C).

### Predicted Biological Processes Regulated by Altered Gut Microbiota

**FIGURE 5:**
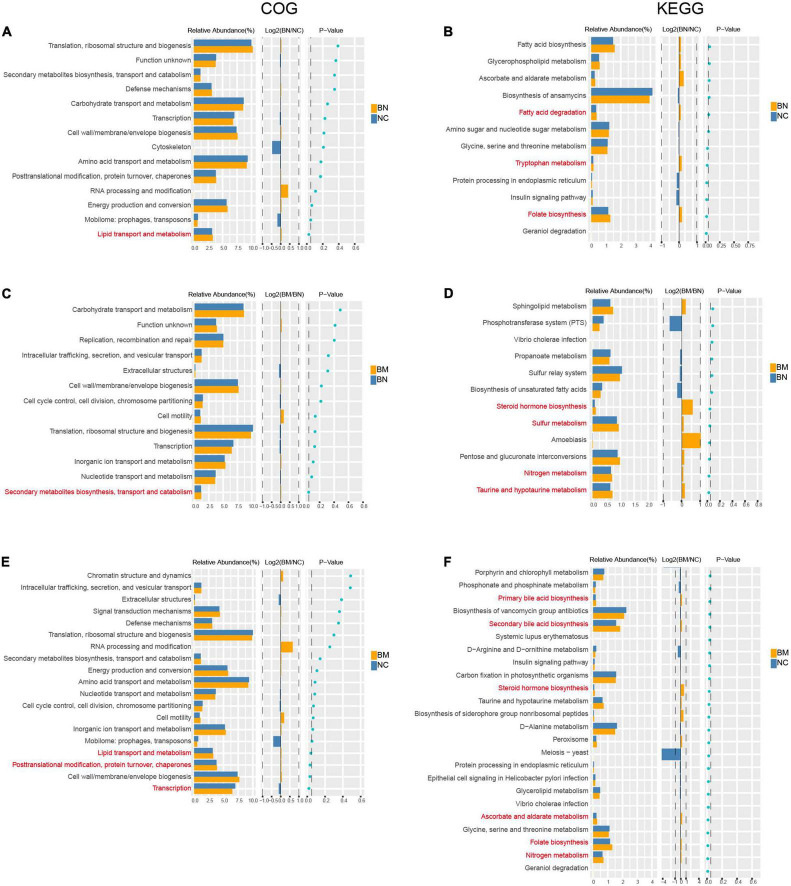
Predicted biological processes mediated by differential gut microbiota based on COG and KEGG analysis. **(A,B)** COG and KEGG analyses of the differential biological processes caused by distinct gut microbiota profiles between NCs and BNs. **(C,D)** COG and KEGG analyses of the differential biological processes caused by distinct gut microbiota profiles between BNs and BMs. **(E,F)** COG and KEGG analyses of the differential biological processes caused by distinct gut microbiota profiles between NCs and BMs. *P* < 0.05 is considered significantly different.

To confirm whether the distinct gut microbial patterns of NCs, BNs, and BMs contributed to the occurrence and metastasis of breast cancer, we performed a phylogenetic investigation of communities by reconstruction of unobserved states (PICRUSt) analysis to predict the significantly different Clusters of Orthologous Groups of proteins (COGs) and Kyoto Encyclopedia of Genes and Genomes (KEGG) pathways. We identified 2 COG and 8 KEGG pathways as significantly different between the BN and NC groups, and the BN group showed increased activities in lipid transport and metabolism, folate biosynthesis, tryptophan metabolism, and fatty acid degradation (Figures 5A,B). The numbers of significantly different COG and KEGG pathways were 1 and 6, respectively, between the BM and BN groups. The BM group showed higher activities in secondary metabolite biosynthesis, transport and catabolism, taurine and hypotaurine metabolism, nitrogen metabolism, sulfur metabolism, and steroid hormone biosynthesis (Figures 5C,D), indicating higher metabolic activities in the gut microbiota in breast cancer patients with bone metastasis than in those without metastasis. Finally, we compared the differential COG and KEGG pathways between the BM and NC groups and identified 5 and 30 differential COG and KEGG pathways, respectively. Various metabolic pathways related to lipid, nitrogen, folate, ascorbate, steroid hormone biosynthesis, and bile acid metabolism and synthesis were upregulated in the BM compared with those in the NC group. Additionally, transcription and posttranslational modification, protein turnover, and chaperones were significantly different between the BM and NC groups (Figures 5E,F). The detailed results of the PICRUSt analysis are shown in Supplementary Table 5.

## Discussion

Breast cancer is the most prevalent malignancy among women, with an estimated 228,155 new cases worldwide in 2021, as well as 43,600 deaths (Siegel et al., 2021). Various effective treatments for breast cancer have been developed in recent years, such as metronomic therapy (Cazzaniga et al., 2019), antibody–drug conjugation systems (Bouchard et al., 2014), nanoparticles and micelles (Hussain et al., 2018), and targeting breast cancer stem cells (Sun et al., 2016), which have contributed to decreased death rates and improved overall survival. Despite developments in breast cancer treatment, the poor prognosis of breast cancer patients with bone metastases remains unchanged. The most common site of breast cancer metastases is bone, and bone metastases in breast cancer usually result in skeletal-related events, including humoral hypercalcemia of malignancy, pathological fractures, spinal cord compression, and pain (Cleeland et al., 2016). The median overall survival (OS) from bone metastasis diagnosis is 40 months in BC patients (Kuchuk et al., 2013), making it critical to develop novel diagnostic and therapeutic methods for breast cancer bone metastases. Recent studies have reported that the gut microbiota participates in the occurrence and progression of breast cancer (Luu et al., 2017; Frugé et al., 2020), and probiotics and prebiotics have been proven to be effective in suppressing the growth of breast cancer (Lakritz et al., 2014; Zengul et al., 2021). However, whether the gut microbiota is involved in breast cancer bone metastases remains largely unknown. The present study comprehensively explored the role of the gut microbiota in breast cancer occurrence and bone metastasis, aiming to enlighten future research on the diagnosis and treatment of breast cancer.

Human bodies possess trillions of microorganisms, and the immense storage of the microbial genome likely significantly affects various biological and pathological processes. Because of advances in high-throughput technology, the microbial profiles can now be detected by 16S rRNA sequencing. Fecal samples from NC, BN, and BM subjects were collected for 16S rRNA sequencing. For gut microbiota comparison, the clinical characteristics of the subjects, including age, antibiotic usage, treatment history, and percentage of hormonal receptor positivity, were comparable among the three groups. The dominant phyla were Bacteroidetes, Firmicutes, Proteobacteria, and Fusobacteria, consistent with findings in a previous study profiling the gut microbiota composition (Bik et al., 2006). These results indicated that the sample collection was reliable and that the discrepancy among the three groups was not caused by different features of the subjects or sample contamination.

Previous studies have demonstrated that gut microbiota dysbiosis contributes to carcinogenesis, including colorectal cancer, lung cancer, prostate cancer, and breast cancer (Picardo et al., 2019). Regarding breast cancer, several studies have reported that breast cancer patients harbor gut microbiota with altered diversity and profiles. Goedert et al. (2018) found that the alpha diversity of the gut microbiota was decreased in postmenopausal breast cancer patients, and altered gut microbiota profiles of both IgA-positive and IgA-negative patients were also detected in the fecal samples of breast cancer patients. According to our study, reduced alpha diversity of the gut microbiota was also detected in BN subjects, although it did not reach significance. Furthermore, consistent with a previous study, PLS-DA showed distinct microbiota profiles between the BN and NC groups. We postulated that gut microbiota homeostasis is indispensable for defending against carcinogenesis and that dysbiosis and an altered microbiome interrupt normal metabolism and immune defense. LEfSe analysis and cladograms were used to analyze the signature microbiota in breast cancer patients. LDA was used to estimate the difference in the effect size of each taxon among the NC, BN, and BM groups, and LDA scores higher than 2 or lower than –2 were considered significantly different. In LDA, *Streptococcus*, *Campylobacter*, and Moraxellaceae were taxa with significantly higher abundances in the BN and BM groups than in the NC group. The elevated abundance of *Streptococcus* in the gut microbiota is related to gastric cancer occurrence and liver metastasis (Yu et al., 2021). Although no evidence supports a direct relationship between *Campylobacter* and breast cancer, *Campylobacter* is related to esophageal cancer occurrence (Poosari et al., 2021). The commonly increased taxa *Streptococcus*, *Campylobacter*, and Moraxellaceae in BN and BM subjects could be risk factors for breast cancer occurrence.

Bone is the third most common site of metastasis for solid tumors, including breast, lung, and colorectal cancer, among which breast cancer accounts for 36% of cancer patients with bone metastasis (Hernandez et al., 2018). In addition to causing pain and skeletal-related events in patients, bone metastasis results in a poor prognosis and shorter overall survival and is rarely cured. Bone is the “soil” for metastasis, and disruption of bone homeostasis can create a more favorable environment for the “seeds,” that is, the disseminated tumor cells. Recently, the regulation of the bone microenvironment by gut microbiota has drawn increased attention. Schwarzer et al. (2016) reported that GF mice showed growth retardation because of reduced levels of IGF-1 and, consequently, a reduced bone mass. Additionally, GF mice have a decreased frequency of CD4 (+) T cells (Sjogren et al., 2012) and increased levels of tumor necrosis factor-alpha and receptor activator of nuclear factor kappa-B ligand in the bone (Ohlsson et al., 2017), indicating the interaction of the gut microbiota with bone by regulating the immune response. Thus, we propose that the gut microbiota regulates breast cancer bone metastasis. Our results showed that the alpha diversity was significantly decreased in the NC, BN, and BM groups, with the most distorted gut microbiota in BM patients, consistent with a previous study reporting that the pre-established disruption of commensal homeostasis in breast cancer mice results in enhanced circulating tumor cells (Buchta Rosean et al., 2019). Because the previous study in mice and our results both implied that the decreased diversity promotes bone metastasis of breast cancer, we postulated that the lack of protective gut microbiota in the BM group compared with those in the NC and BN groups accelerates bone metastasis. According to LDA, the high-abundance taxa in the NC and BN groups were similar to those in the BM group. *Megamonas* and *Akkermansia* showed significantly higher abundance in both the NC and BN groups. Ubachs et al. (2021) demonstrated that *Megamonas* was decreased in the feces of cachectic cancer patients; in recurrent pancreatic tumor tissues, *Akkermansia* was decreased (Jeong et al., 2020). Additionally, Ren et al. (2020) developed the nanoparticle NpRg3 to inhibit hepatocellular carcinoma development and metastasis by elevating the abundance of Verrucomicrobia. The above results and previous studies support that *Megamonas* and *Akkermansia* can be diagnostic indexes and therapeutic targets for bone metastasis.

In addition to exploring the gut microbiota in regulating breast cancer occurrence and bone metastasis, we used PICRUSt analysis to predict the biological and pathological processes affected by the distinct microbial profiles among the NC, BN, and BM groups. The common upregulated terms in the BM and BN groups were lipid transportation and metabolism as well as folate biosynthesis. Dysregulation of lipid metabolism is common in breast cancer, and several studies have reported that lipids can be indexes for breast cancer diagnosis and prognosis evaluation (Guo et al., 2020). Zhu et al. (2016) demonstrated that sterol regulatory-element binding protein –1-mediated transcription of lipogenic genes and lipid production are essential for breast cancer development. However, Coleman et al. (2021) reported that folate deprivation or anti-folate therapy inhibits triple-negative breast cancer cell growth by inducing mitochondrial dysfunction. The enhanced lipid transport and metabolism and folate biosynthesis caused by the gut microbiota in the BN and BM groups may participate in breast cancer occurrence.

The common terms enriched in the BM group compared with those in the NC or BN group should be the most likely processes influencing breast cancer bone metastasis. Steroid hormone biosynthesis was significantly upregulated in the BM group, as predicted by PICURSt analysis. Sex steroid levels have long been related to higher breast cancer risk. The breast cancer risk is almost double in subjects with circulating sex steroid levels in the highest quintile compared with those in the lowest quintile, with a hazard ratio of 2.15 [95% confidence interval (CI), 1.87-2.46] for estradiol, 1.81 (95% CI, 1.5-2.10) for estrone, and 2.04 (95% CI, 1.76-2.37) for testosterone (Picon-Ruiz et al., 2017). Li et al. (2016) elucidated that in GF mice, sex steroid deficiency failed to induce trabecular bone loss, and the probiotic *Lactobacillus rhamnosus* protected against bone loss in normal mice caused by sex steroid deficiency. Furthermore, Flores et al. (2012) reported that the richness and functions of the gut microbiota, including but not limited to β-glucuronidase, influence non-ovarian estrogen levels and affect the risk of estrogen-related conditions. The complex interactions among microbiota, steroids, and bone support that the gut microbiota may regulate steroid hormone levels and thus participate in breast cancer bone metastasis.

The present study needs further improvement. By collecting fecal samples of NCs, BNs, and BMs, we identified the signature gut microbiota involved in breast cancer occurrence and bone metastasis. However, the relatively small sample size restricts further investigation of the causative roles of the signature gut microbiota in breast cancer occurrence and bone metastasis. The present study is the first to report the relationship between gut microbiota and breast cancer metastasis, providing new insights into integrating gut microbiota interference, such as antibiotic or probiotic administration, with regular treatments to help delay or prevent the development of breast cancer. However, some unsolved questions must be mentioned. What are the intrinsic mechanisms by which the signature gut microbiota regulate breast cancer occurrence and bone metastasis? Does interference with the gut microbiota contribute to a better prognosis in breast cancer patients? To what extent could the signature gut microbiota predict breast cancer bone metastasis? These are the unsolved questions that warrant further exploration.

## Conclusion

In the present study, we collected fecal samples from NC, BN, and BM subjects; performed a comprehensive gut microbiota analysis, and revealed distinct profiles and decreased community diversity in the order of NCs, BNs, and BMs. Various gut microbiota was identified as correlated with breast cancer occurrence and bone metastasis. *Streptococcus*, *Campylobacter*, and Moraxellaceae, which showed higher abundance in both the BN and BM groups, were considered related to breast cancer occurrence. The lack of *Megamonas* and *Akkermansia* in the BM group compared with those in the NC and BN groups is related to bone metastasis. PICRUSt analysis predicted the biological processes affected by distinct gut microbiota and revealed that lipid transport and metabolism, as well as folate biosynthesis, participate in breast cancer occurrence and that steroid hormone biosynthesis influences bone metastasis. Our study is the first comprehensive analysis of the gut microbiota in NC, BN, and BM subjects and may provide insight into future diagnostic and therapeutic studies in breast cancer occurrence and bone metastasis.

## Data Availability Statement

The datasets presented in this study can be found in online repositories. The names of the repository/repositories and accession number(s) can be found in the article/Supplementary Material. The data are available in the SRA database, accession number PRJNA804967.

## Ethics Statement

The studies involving human participants were reviewed and approved by the Ethics Committee of the Sun Yat-sen Memorial Hospital, Sun Yat-sen University. The patients/participants provided their written informed consent to participate in this study.

## Author Contributions

SH, WY, and WP supervised the project. YW, XZ, and CK designed and wrote the manuscript. YW performed the bioinformatics analysis. XZ and CK recruited the subjects. CZ and LJ extracted microbial DNA from the fecal samples. MM, SZ, and CY collected the fecal samples and ran the specimen bank. All authors read and approved the final manuscript.

## Conflict of Interest

The authors declare that the research was conducted in the absence of any commercial or financial relationships that could be construed as a potential conflict of interest.

## Publisher’s Note

All claims expressed in this article are solely those of the authors and do not necessarily represent those of their affiliated organizations, or those of the publisher, the editors and the reviewers. Any product that may be evaluated in this article, or claim that may be made by its manufacturer, is not guaranteed or endorsed by the publisher.

## References

[B1] Allemani C. Matsuda T. Di Carlo V. Harewood R. Matz M. Nikšić M. (2018). Global surveillance of trends in cancer survival 2000–14 (CONCORD-3): analysis of individual records for 37 513 025 patients diagnosed with one of 18 cancers from 322 population-based registries in 71 countries. *Lancet* 391 1023–1075. 10.1016/S0140-6736(17)33326-3 29395269 PMC5879496

[B2] Bik E. M. Eckburg P. B. Gill S. R. Nelson K. E. Purdom E. A. Francois F. (2006). Molecular analysis of the bacterial microbiota in the human stomach. *Proc. Natl. Acad. Sci. U.S.A.* 103 732–737. 10.1073/pnas.0506655103 16407106 PMC1334644

[B3] Bouchard H. Viskov C. Garcia-Echeverria C. (2014). Antibody-drug conjugates-a new wave of cancer drugs. *Bioorgan. Med. Chem. Lett.* 24 5357–5363. 10.1016/j.bmcl.2014.10.021 25455482

[B4] Brook N. Brook E. Dharmarajan A. Dass C. R. Chan A. (2018). Breast cancer bone metastases: pathogenesis and therapeutic targets. *Int. J. Biochem. Cell Biol.* 96 63–78. 10.1016/j.biocel.2018.01.003 29309917

[B5] Buchta Rosean C. Bostic R. R. Ferey J. C. M. Feng T. Y. Azar F. N. Tung K. S. (2019). Preexisting commensal dysbiosis is a host-intrinsic regulator of tissue inflammation and tumor cell dissemination in hormone receptor-positive breast cancer. *Cancer Res.* 79 3662–3675. 10.1158/0008-5472.CAN-18-3464 31064848 PMC6983951

[B6] Caporaso J. G. Kuczynski J. Stombaugh J. Bittinger K. Bushman F. D. Costello E. K. (2010). QIIME allows analysis of high-throughput community sequencing data. *Nat. Methods* 7 335–336. 10.1038/nmeth.f.303 20383131 PMC3156573

[B7] Caporaso J. G. Lauber C. L. Walters W. A. Berg-Lyons D. Huntley J. Fierer N. (2012). Ultra-high-throughput microbial community analysis on the Illumina HiSeq and MiSeq platforms. *ISME J.* 6 1621–1624. 10.1038/ismej.2012.8 22402401 PMC3400413

[B8] Cazzaniga M. E. Biganzoli L. Cortesi L. De Placido S. Donadio M. Fabi A. (2019). Treating advanced breast cancer with metronomic chemotherapy: what is known, what is new and what is the future? *Onco Targets Ther.* 12 2989–2997. 10.2147/OTT.S189163 31114242 PMC6485034

[B9] Cleeland C. von Moos R. Walker M. S. Wang Y. Gao J. Chavez-MacGregor M. (2016). Burden of symptoms associated with development of metastatic bone disease in patients with breast cancer. *Support. Care Cancer Off. J. Multinatl. Assoc. Support. Care Cancer* 24 3557–3565. 10.1007/s00520-016-3154-x 27022965 PMC4917575

[B10] Coleman M. F. O’Flanagan C. H. Pfeil A. J. Chen X. Pearce J. B. Sumner S. (2021). Metabolic response of triple-negative breast cancer to folate restriction. *Nutrients* 13:1637. 10.3390/nu13051637 34068120 PMC8152779

[B11] D’Amelio P. Sassi F. (2018). Gut microbiota, immune system, and bone. *Calcif Tissue Int.* 102 415–425. 10.1007/s00223-017-0331-y 28965190

[B12] Edgar R. C. (2010). Search and clustering orders of magnitude faster than BLAST. *Bioinformatics (Oxford, England)* 26 2460–2461. 10.1093/bioinformatics/btq461 20709691

[B13] Edgar R. C. (2013). UPARSE: highly accurate OTU sequences from microbial amplicon reads. *Nat. Methods* 10 996–998. 10.1038/nmeth.2604 23955772

[B14] Edgar R. C. Haas B. J. Clemente J. C. Quince C. Knight R. (2011). UCHIME improves sensitivity and speed of chimera detection. *Bioinformatics (Oxford, England)* 27 2194–2200. 10.1093/bioinformatics/btr381 21700674 PMC3150044

[B15] Ferlay J. Soerjomataram I. Dikshit R. Eser S. Mathers C. Rebelo M. (2015). Cancer incidence and mortality worldwide: sources, methods and major patterns in GLOBOCAN 2012. *Int. J. Cancer* 136 E359–E386. 25220842 10.1002/ijc.29210

[B16] Flores R. Shi J. Fuhrman B. Xu X. Veenstra T. D. Gail M. H. (2012). Fecal microbial determinants of fecal and systemic estrogens and estrogen metabolites: a cross-sectional study. *J. Transl. Med.* 10:253. 10.1186/1479-5876-10-253 23259758 PMC3552825

[B17] Frugé A. D. Van der Pol W. Rogers L. Q. Morrow C. D. Tsuruta Y. Demark-Wahnefried W. (2020). Fecal Akkermansia muciniphila is associated with body composition and microbiota diversity in overweight and obese women with breast cancer participating in a presurgical weight loss trial. *J. Acad. Nutr. Dietetics* 120 650–659. 10.1016/j.jand.2018.08.164 30420171 PMC6509025

[B18] Goedert J. J. Hua X. Bielecka A. Okayasu I. Milne G. L. Jones G. S. (2018). Postmenopausal breast cancer and oestrogen associations with the IgA-coated and IgA-noncoated faecal microbiota. *Br. J. Cancer* 118 471–479. 10.1038/bjc.2017.435 29360814 PMC5830593

[B19] Guo R. Chen Y. Borgard H. Jijiwa M. Nasu M. He M. (2020). The function and mechanism of lipid molecules and their roles in the diagnosis and prognosis of breast cancer. *Molecules (Basel, Switzerland)* 25:4864. 10.3390/molecules25204864 33096860 PMC7588012

[B20] Hernandez C. J. Guss J. D. Luna M. Goldring S. R. (2016). Links between the microbiome and bone. *J. Bone Miner. Res.* 31 1638–1646. 10.1002/jbmr.2887 27317164 PMC5434873

[B21] Hernandez R. K. Wade S. W. Reich A. Pirolli M. Liede A. Lyman G. H. (2018). Incidence of bone metastases in patients with solid tumors: analysis of oncology electronic medical records in the United States. *BMC Cancer* 18:44. 10.1186/s12885-017-3922-0 29306325 PMC5756362

[B22] Hussain Z. Khan J. A. Murtaza S. (2018). Nanotechnology: an emerging therapeutic option for breast cancer. *Crit. Rev. Eukaryotic Gene Expr.* 28 163–175. 10.1615/CritRevEukaryotGeneExpr.2018022771 30055543

[B23] Jeong J. Y. Kim T. B. Kim J. Choi H. W. Kim E. J. Yoo H. J. (2020). Diversity in the extracellular vesicle-derived microbiome of tissues according to tumor progression in pancreatic cancer. *Cancers* 12:2346. 10.3390/cancers12092346 32825137 PMC7563179

[B24] Kuchuk I. Hutton B. Moretto P. Ng T. Addison C. L. Clemons M. (2013). Incidence, consequences and treatment of bone metastases in breast cancer patients-experience from a single cancer centre. *J. Bone Oncol.* 2 137–144. 10.1016/j.jbo.2013.09.001 26909284 PMC4723382

[B25] Lakritz J. R. Poutahidis T. Levkovich T. Varian B. J. Ibrahim Y. M. Chatzigiagkos A. (2014). Beneficial bacteria stimulate host immune cells to counteract dietary and genetic predisposition to mammary cancer in mice. *Int. J. Cancer* 135 529–540. 10.1002/ijc.28702 24382758 PMC4131439

[B26] Li J. Y. Chassaing B. Tyagi A. M. Vaccaro C. Luo T. Adams J. (2016). Sex steroid deficiency-associated bone loss is microbiota dependent and prevented by probiotics. *J. Clin. Investigat.* 126 2049–2063. 10.1172/JCI86062 27111232 PMC4887186

[B27] Li R. Zhou R. Wang H. Li W. Pan M. Yao X. (2019). Gut microbiota-stimulated cathepsin K secretion mediates TLR4-dependent M2 macrophage polarization and promotes tumor metastasis in colorectal cancer. *Cell Death Differ.* 26 2447–2463. 10.1038/s41418-019-0312-y 30850734 PMC6889446

[B28] Luu T. H. Michel C. Bard J. M. Dravet F. Nazih H. Bobin-Dubigeon C. (2017). Intestinal proportion of Blautia sp. is associated with clinical stage and histoprognostic grade in patients with early-stage breast cancer. *Nutr. Cancer* 69 267–275. 10.1080/01635581.2017.1263750 28094541

[B29] Ma J. Li J. Wang Y. Chen W. Zheng P. Chen Y. (2020). WSZG inhibits BMSC-induced EMT and bone metastasis in breast cancer by regulating TGF-beta1/Smads signaling. *Biomed. Pharmacother.* 121:109617. 10.1016/j.biopha.2019.109617 31810139

[B30] Magoč T. Salzberg S. L. (2011). FLASH: fast length adjustment of short reads to improve genome assemblies. *Bioinformatics (Oxford, England)* 27 2957–2963. 10.1093/bioinformatics/btr507 21903629 PMC3198573

[B31] Matson V. Fessler J. Bao R. Chongsuwat T. Zha Y. Alegre M. L. (2018). The commensal microbiome is associated with anti-PD-1 efficacy in metastatic melanoma patients. *Science (New York, NY)* 359 104–108. 10.1126/science.aao3290 29302014 PMC6707353

[B32] Mayer E. A. Tillisch K. Gupta A. (2015). Gut/brain axis and the microbiota. *J. Clin. Invest.* 125 926–938. 25689247 10.1172/JCI76304 PMC4362231

[B33] McKee A. M. Kirkup B. M. Madgwick M. Fowler W. J. Price C. A. Dreger S. A. (2021). Antibiotic-induced disturbances of the gut microbiota result in accelerated breast tumor growth. *iScience* 24:103012. 10.1016/j.isci.2021.103012 34522855 PMC8426205

[B34] Morrison D. J. Preston T. (2016). Formation of short chain fatty acids by the gut microbiota and their impact on human metabolism. *Gut Microbes* 7 189–200. 10.1080/19490976.2015.1134082 26963409 PMC4939913

[B35] Ohlsson C. Nigro G. Boneca I. G. Bäckhed F. Sansonetti P. Sjögren K. (2017). Regulation of bone mass by the gut microbiota is dependent on NOD1 and NOD2 signaling. *Cell. Immunol.* 317 55–58. 10.1016/j.cellimm.2017.05.003 28576260

[B36] Picardo S. L. Coburn B. Hansen A. R. (2019). The microbiome and cancer for clinicians. *Crit. Rev. Oncol. Hematol.* 141 1–12. 10.1016/j.critrevonc.2019.06.004 31202124

[B37] Picon-Ruiz M. Morata-Tarifa C. Valle-Goffin J. J. Friedman E. R. Slingerland J. M. (2017). Obesity and adverse breast cancer risk and outcome: mechanistic insights and strategies for intervention. *CA Cancer J. Clin.* 67 378–397. 10.3322/caac.21405 28763097 PMC5591063

[B38] Poosari A. Nutravong T. Sa-Ngiamwibool P. Namwat W. Chatrchaiwiwatana S. Ungareewittaya P. (2021). Association between infection with Campylobacter species, poor oral health and environmental risk factors on esophageal cancer: a hospital-based case-control study in Thailand. *Eur. J. Med. Res.* 26:82. 10.1186/s40001-021-00561-3 34332608 PMC8325836

[B39] Quail D. F. Olson O. C. Bhardwaj P. Walsh L. A. Akkari L. Quick M. L. (2017). Obesity alters the lung myeloid cell landscape to enhance breast cancer metastasis through IL5 and GM-CSF. *Nat. Cell Biol.* 19 974–987. 10.1038/ncb3578 28737771 PMC6759922

[B40] Ren Z. Chen X. Hong L. Zhao X. Cui G. Li A. (2020). Nanoparticle conjugation of Ginsenoside Rg3 inhibits hepatocellular carcinoma development and metastasis. *Small (Weinheim an der Bergstrasse, Germany)* 16:e1905233. 10.1002/smll.201905233 31814271

[B41] Schloss P. D. Westcott S. L. Ryabin T. Hall J. R. Hartmann M. Hollister E. B. (2009). Introducing mothur: open-source, platform-independent, community-supported software for describing and comparing microbial communities. *Appl. Environ. Microbiol.* 75 7537–7541. 10.1128/AEM.01541-09 19801464 PMC2786419

[B42] Schwarzer M. Makki K. Storelli G. Machuca-Gayet I. Srutkova D. Hermanova P. (2016). Lactobacillus plantarum strain maintains growth of infant mice during chronic undernutrition. *Science (New York, NY)* 351 854–857. 10.1126/science.aad8588 26912894

[B43] Siegel R. L. Miller K. D. Fuchs H. E. Jemal A. (2021). Cancer statistics, 2021. *CA Cancer J. Clin.* 71 7–33. 33433946 10.3322/caac.21654

[B44] Sjogren K. Engdahl C. Henning P. Lerner U. H. Tremaroli V. Lagerquist M. K. (2012). The gut microbiota regulates bone mass in mice. *J. Bone Miner. Res.* 27 1357–1367. 10.1002/jbmr.1588 22407806 PMC3415623

[B45] Sun R. Shen S. Zhang Y. J. Xu C. F. Cao Z. T. Wen L. P. (2016). Nanoparticle-facilitated autophagy inhibition promotes the efficacy of chemotherapeutics against breast cancer stem cells. *Biomaterials* 103 44–55. 10.1016/j.biomaterials.2016.06.038 27376558

[B46] Terrisse S. Derosa L. Iebba V. Ghiringhelli F. Vaz-Luis I. Kroemer G. (2021). Intestinal microbiota influences clinical outcome and side effects of early breast cancer treatment. *Cell Death Differ.* 28 2778–2796. 10.1038/s41418-021-00784-1 33963313 PMC8408230

[B47] Ubachs J. Ziemons J. Soons Z. Aarnoutse R. van Dijk D. P. J. Penders J. (2021). Gut microbiota and short-chain fatty acid alterations in cachectic cancer patients. *J. Cachexia Sarcopenia Muscle* 12 2007–2021. 10.1002/jcsm.12804 34609073 PMC8718054

[B48] Wilkinson T. J. Huws S. A. Edwards J. E. Kingston-Smith A. H. Siu-Ting K. Hughes M. (2018). CowPI: a rumen microbiome focussed Version of the PICRUSt functional inference software. *Front. Microbiol.* 9:1095. 10.3389/fmicb.2018.01095 29887853 PMC5981159

[B49] Yu D. Yang J. Jin M. Zhou B. Shi L. Zhao L. (2021). Fecal streptococcus alteration is associated with gastric cancer occurrence and liver metastasis. *mBio* 12:e0299421. 10.1128/mBio.02994-21 34872346 PMC8649758

[B50] Zengul A. G. Demark-Wahnefried W. Barnes S. Morrow C. D. Bertrand B. Berryhill T. F. (2021). Associations between dietary fiber, the fecal microbiota and estrogen metabolism in postmenopausal women with breast cancer. *Nutr. Cancer* 73 1108–1117. 10.1080/01635581.2020.1784444 32590914 PMC7875566

[B51] Zhu J. Liao M. Yao Z. Liang W. Li Q. Liu J. (2018). Breast cancer in postmenopausal women is associated with an altered gut metagenome. *Microbiome* 6:136. 10.1186/s40168-018-0515-3 30081953 PMC6080540

[B52] Zhu Z. Zhao X. Zhao L. Yang H. Liu L. Li J. (2016). p54(nrb)/NONO regulates lipid metabolism and breast cancer growth through SREBP-1A. *Oncogene* 35 1399–1410. 10.1038/onc.2015.197 26148231

