# Supplementary material for: Variations in the Gut Microbiota in Breast Cancer Occurrence and Bone Metastasis
Source: Front Microbiol. 2022 May 26;13:894283. doi: 10.3389/fmicb.2022.894283 (PMC9204246; doi:10.3389/fmicb.2022.894283)
Supplement: Supplementary file 1 [file Data_Sheet_1.docx]

Supplementary Material

**Supplementary Figure 1.** **OTU cumulative curve of all the 16S rRNA sequencing fecal samples**


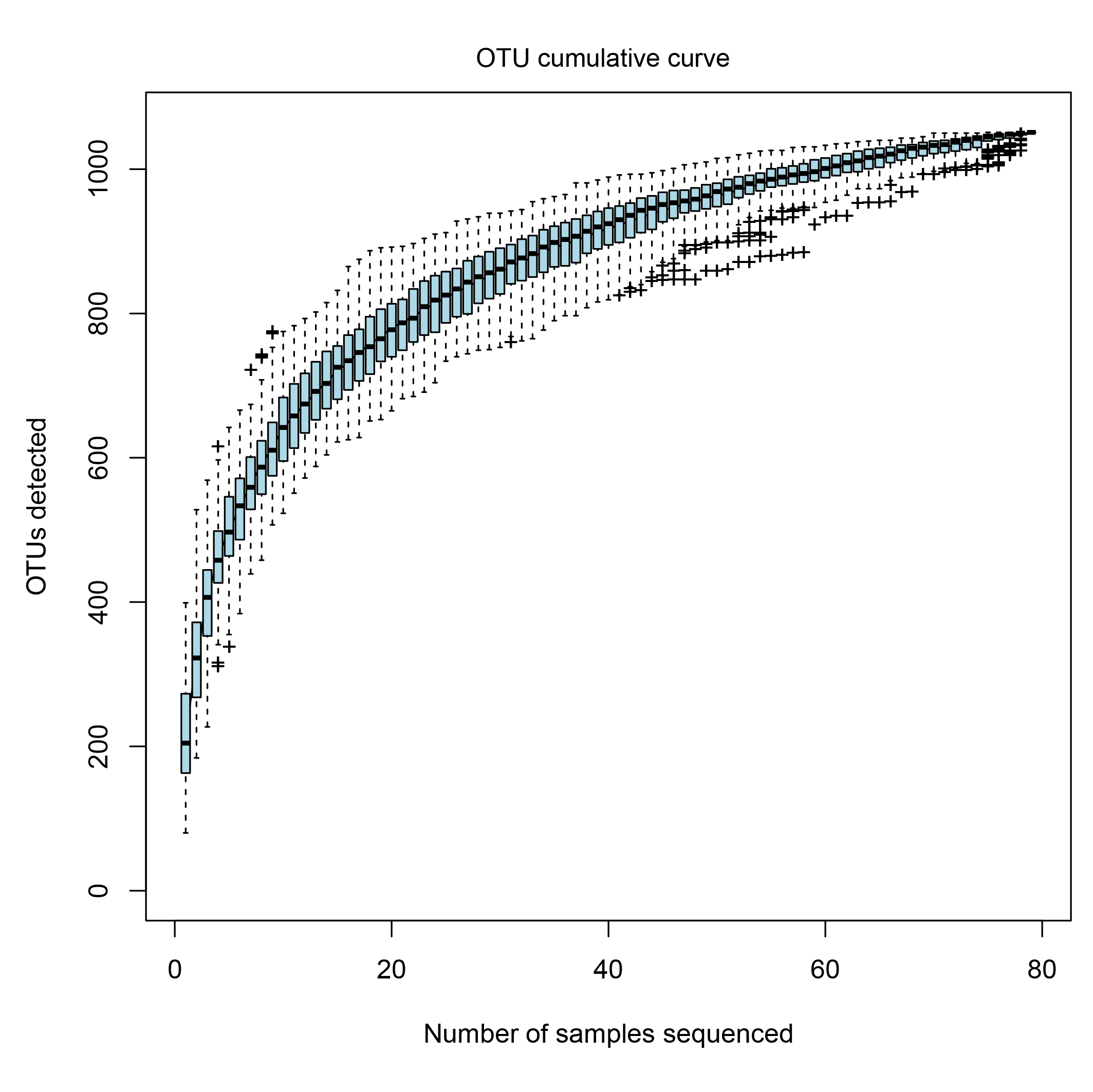

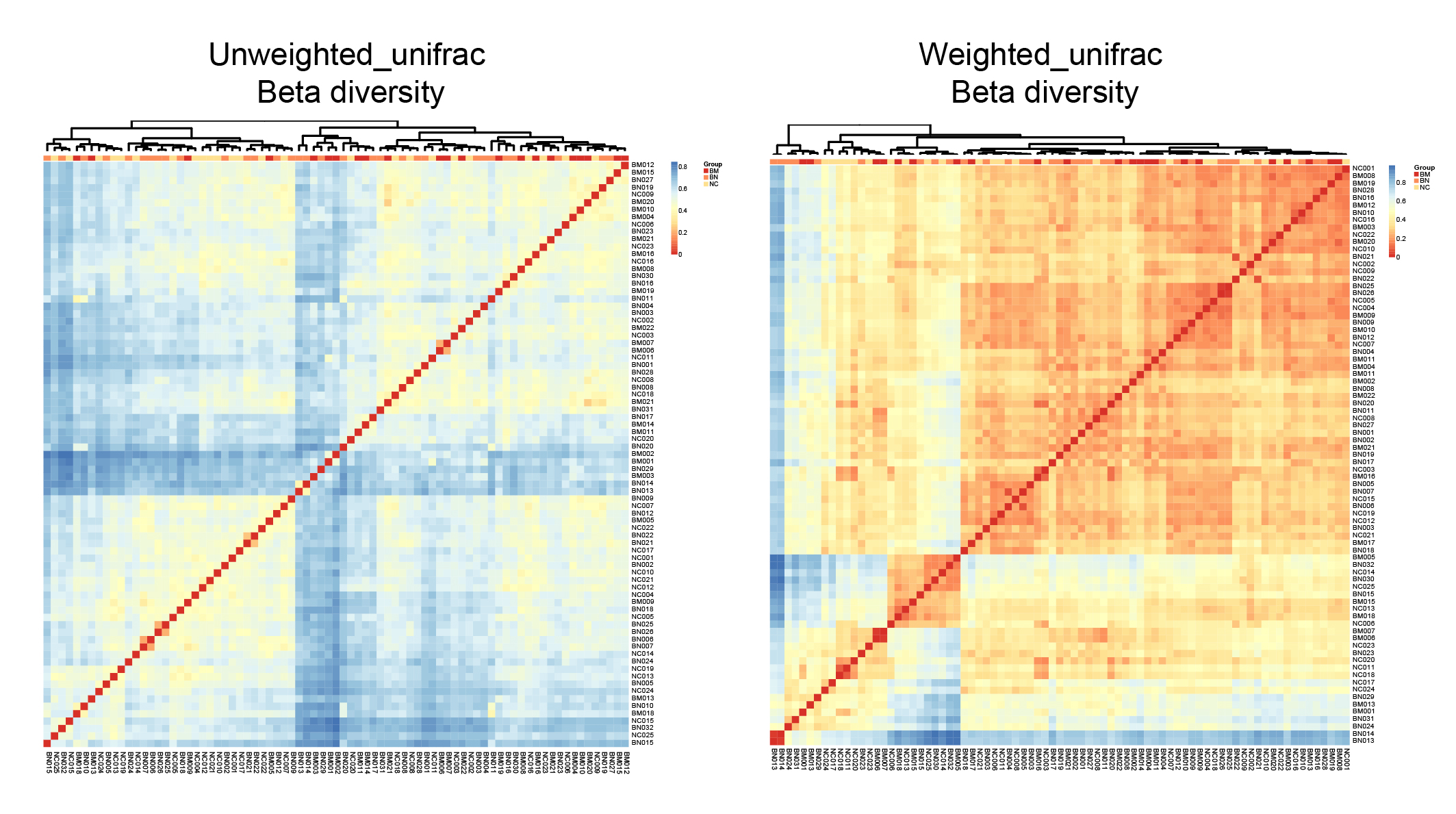


**Supplementary Figure 2. Heatmaps showing the unweighted and weighted unifrac beta diversity clustering of all the 16S rRNA sequencing fecal samples**

**Table S1 Clinical characteristics of included subjects**

**Table S2 Quality control statistics of the 16S rRNA sequencing**

**Table S3 Gut microbial composition profiles**

**Table S4 Microbial structures in LEfSe analysis**

**Table S5 The predicted differential COG and KEGG pathways by PICRUSt analysis**
